# Detection of Carbapenem-Resistant Enterobacterales in Simulated Blood Culture in 15 Minutes

**DOI:** 10.3390/life11020145

**Published:** 2021-02-14

**Authors:** Daria Baer, Maya Azrad, Nora Saleh, Avi Peretz

**Affiliations:** 1 The Azrieli Faculty of Medicine, Bar-Ilan University, Safed 1311502, Israel; daria.baer@live.biu.ac.il; 2 Clinical Microbiology Laboratory, The Baruch Padeh Medical Center, Poriya, Tiberias 1528001, Israel; mazrad@poria.health.gov.il (M.A.); nosaleh@poria.health.gov.il (N.S.)

**Keywords:** bacteremia, carbapenem-resistant Enterobacterales, NG-Test CARBA 5, bloodstream infections, blood culture

## Abstract

Bacteremia leading to sepsis and organ dysfunction is a life-threatening situation, leading to death of up to one fourth of the infected individuals around the world. One major challenge in the treatment of sepsis is the rising prevalence of antibiotic resistant bacteria, such as carbapenem-resistant Enterobacterales (CRE). In recent years, several molecular assays have been developed for the detection of CRE mechanisms, enabling rapid results reporting. We evaluated the performance of the NG-Test CARBA 5 (NG Biotech) kit in detection of CRE in simulated blood cultures. Carbapenemase-producing (CP) CRE isolates (*n* = 38) and non-carbapenemase CRE (Non-CP) isolates (*n* = 10), previously identified using the routine methods practiced at the clinical microbiology laboratory of the Baruch Padeh Medical Center, Israel, were used in this analysis. Variable concentrations of the bacterial isolates were added to a suspension composed of human blood and saline, simulating the composition of a blood culture. Samples were then transferred to an anaerobic blood culture bottle and later tested with the NG-Test CARBA 5 (NG Biotech) kit, that identifies the CRE mechanism within 15 min. The NG-Test CARBA 5 kit correctly identified 43 samples (89.5%). The sensitivity and specificity of the kits were 86.8% and 100%, respectively. In conclusion, the NG-Test CARBA 5 kit is a reliable and accessible tool for the rapid diagnosis of CRE bloodstream infections.

## 1. Introduction

Bloodstream infections (BSI) are a leading cause of morbidity and mortality worldwide [1,2]. Diagnosis of BSI is primarily blood culture (BC)-based, involving injection of patient blood into a bottle containing liquid culture medium, which is then placed into a designated incubator that monitors bacterial growth by means of CO_2_ measurements [3]. When bacterial growth is identified, the laboratory staff samples the respective BC bottle, performs Gram staining and inoculates the sample on solid agar medium, together resulting in the prolonged turnaround time of pathogen identification in BSI (at least 24–48 h, depending on the organism). Furthermore, antibiotic susceptibility testing (AST), which can only be performed after the growth of the pathogen colonies on solid medium, demands approximately 24 h. Each delay in pathogen identification and antibiotic treatment adjustment has serious clinical implications, particularly with regards to infections with multidrug resistant bacteria, such as carbapenem-resistant Enterobacterales (CRE) [4,5,6]. Therefore, rapid assays for pathogen identification in BSI are needed.

CRE are Gram-negative bacteria that are resistant to carbapenems, a class of antibiotics used to treat severe infections, as well as to most other antibiotics commonly used today [7]. CREs are classified as carbapenemase-producing CREs (CP-CREs) or non-CP-CREs, based on their ability to produce carbapenemases, the enzymes that hydrolyze the antibiotic. There are different carbapenemase enzymes, namely, *Klebsiella pneumoniae* carbapenemase (KPC), New Delhi metallo-beta-lactamase (NDM), carbapenem-hydrolyzing oxacillinases (e.g., OXA-48-like), Verona integron-encoded metallo-β-lactamase (VIM) and imipenemase metallo-β-lactamase (IMP) [7].

In recent years, various rapid pathogen detection assays, namely, FilmArray blood culture identification (BCID) assay (BioFire Diagnostics, Salt Lake City, UT, USA) and Verigene Gram-negative blood culture (BC-GN) assay (Nanosphere, Northbrook, IL, USA), have been developed and tested for BC sampling [8]. These tests are mainly based on molecular biology methods, require dedicated equipment, and incur extra costs. Another recent development involves rapid bacterial antigen identification, which serves mainly for the identification of drug-resistant bacteria and circumvents the need for molecular methods. In particular, the NG-Test CARBA 5 kit (NG Biotech, Guipry, France) is an immunochromatographic assay allowing for determination of the CRE resistance mechanism, all within 15 min [9]. Originally, the NG-Test CARBA 5 kit was developed for detection of carbapenemases in isolated colonies. Recently, the assay was demonstrated to perform well for direct detection of CRE bacteria in blood culture, without the need for bacterial cultivation [10]. Several studies have evaluated the reliability of the NG-Test CARBA 5 kit as a diagnostic tool for recognition of CRE in bacterial colonies [10,11]. The current work assessed the kit’s performance and lower limit of detection in blood CRE samples.

## 2. Materials and Methods

### 2.1. Bacterial Isolates

This study was performed in the clinical microbiology laboratory at the Baruch Padeh Medical Center, Israel. Bacterial clinical or screening samples previously found to be resistant to carbapenems using our routine methods, were retested. Out of the 48 isolates, 10 were non-CP CRE and 38 were CP-CRE (1 IMP, 10 NDM, 8 OXA-48, 6 VIM, and 13 KPC) (Table 1).

Briefly, testing involved Enterobacterales identification by matrix-assisted laser desorption ionization-time of flight (MALDI-TOF) technology (Bruker Daltonics, Bremen, Germany), and then using the β CARBA kit (Bio-Rad Laboratories Ltd., Rishon Lezion, Israel), which detects strains with a decreased susceptibility to carbapenems. Colonies that gave a positive result were further analyzed using the Xpert Carba-R (Cepheid, Sunnyvale, CA, USA) polymerase chain reaction (PCR) assay, that detects the five most prevalent carbapenemases (KPC, NDM, VIM, OXA-48, and IMP). In order to demonstrate that bacteria type did not influence kit performance, different types of CRE bacteria were tested, such as *Escherichia coli* and *Klebsiella pneumoniae*. Moreover, bacteria with various types of carbapenemases (NDM, KPC, OXA, VIM, IMP) were examined.

All isolates were stored at –80 °C until analysis. Prior to analysis by the NG-Test kit, the isolates were grown on MacConkey agar (BD Diagnostics, Sparks, MD, USA), at 36 ± 1 °C, in 5% CO_2_, for 24 h.

### 2.2. Simulated Blood Culture Preparation

Suspensions simulating patient blood samples were prepared by diluting human blood with saline, at a ratio of 45% blood unit and 55% saline. Then, a bacterial suspension of each isolate was prepared in saline, to a turbidity of 0.5 McFarland, which is equivalent to 1 × 10^8^–2 × 10^8^ colony-forming units (CFU)/mL. The bacterial suspension was then further diluted, to create variable concentrations of bacteria that were later mixed with the blood suspensions. Blood-bacteria suspensions (9 mL) were introduced to an anaerobic blood culture bottle (Plus Anaerobic/F Culture Vials (BD Diagnostics, Sparks, MD, USA)), producing “blood cultures” with a final concentration of 6.75 × 10^7^ CFU/mL. Samples from each bottle were then tested with the NG-Test CARBA 5 (NG Biotech) kit. The lowest concentration recognized by the NG-Test CARBA 5 kit was determined for each tested pathogen.

### 2.3. Detection of Carbapenemases by the NG-Test CARBA 5 Kit

The NG-Test CARBA 5 kit (NG Biotech, Guipry, France) is a qualitative rapid immunoassay, in which mouse monoclonal antibodies directed against KPC (K), OXA (O), VIM (V), IMP (I), and NDM (N), are immobilized on the nitrocellulose membrane test zones K, O, V, I, and N, respectively. The tested sample is mixed with 150 μL extraction buffer; 100 μL of this mixture is then dispensed into the cassette well and allowed to migrate towards the conjugate pad. If a carbapenemase is present in the suspension, it reacts with labelled monoclonal anti-carbapenemase antibodies. The carbapenemase-antibodies complex migrates through the nitrocellulose membrane and is captured by corresponding monoclonal anti-carbapenemase antibodies immobilized on the membrane, resulting in a red line (or lines) in the test zone (s) and in the control zone.

Samples from each “blood culture” (1 mL) were transferred into an Eppendorf tube, after which, 1 mL lysis buffer (Accuprep Genomic DNA extraction kit, Bioneer, Korea) was added. The Eppendorf tube was vortexed for 20 s and then centrifuged for 1 min at 13,000 rpm. Pellets were then washed with 1 mL wash buffer (Accuprep Genomic DNA extraction kit, Bioneer, Korea), vortexed for 20 s, and centrifuged for 1 min at 13,000 rpm. Pellets were then resuspended in 150 µL extraction buffer (supplied with the kit) and placed on the test zone of the cassette and later analyzed for results.

As mentioned above, for each isolate, we tested several concentrations of isolate colonies per mL “blood culture” in order to define the lower limit of detection. It should be noted that each of the isolates was correctly identified by the kit, when tested in colony form (without preparing a blood-bacteria suspension).

### 2.4. Statistical Analysis

The Xpert Carba-R (Cepheid, Sunnyvale, CA, USA) PCR assay served as the reference method for calculating sensitivity, specificity, negative predictive value (NPV), and positive predictive value (PPV). Therefore, specimens that were found positive or negative by the Xpert Carba-R assay were defined as “True Positive” or “True Negative”, respectively.

Agreement between Xpert Carba-R and the NG-Test CARBA 5 kit was calculated as the percentage of samples that had the same results for both assays, out of the total number of tested samples. Data were analyzed using SAS version 9.3 (SAS Institute, Cary, NC, USA).

## 3. Results

The distribution of the different bacterial isolates according to their carbapenem-resistance mechanism is presented in Table 2. It should be noted that this distribution does not represent the prevalence of the different carbapenemases.

### 3.1. Identification of CRE Mechanism in Simulated Blood Culture

Out of the 48 tested isolates, the NG-Test CARBA 5 kit correctly identified 43 samples (89.5%). Overall, 5 (10.5%) isolates yielded a false negative result, while no isolates generated a false positive or an invalid result (Table 3). The kit identified all the 11 (100%) KPC-producers, 8 of the 10 (80%) NDM-producer isolates, 7 of the 8 (87.5%) OXA-48-producing bacteria, and 5 of the 6 (83.3%) VIM-producers. The one IMP-producer isolate was not detected by the kit. Taken together, the NG-Test CARBA 5 kit’s sensitivity, specificity, NPV, and PPV for the detection of CRE in simulated blood cultures were 89.6%, 100%, 66.7%, and 100%, respectively (Table 4).

### 3.2. Determination of the Average and Lowest Concentrations of Bacteria Identified by the Kit

Table 5 presents the average and lowest concentrations of bacteria in the simulated blood culture identified by the NG-CARBA5 kit, for each resistance mechanism. The lowest average concentration detected by the kit was 3.57 × 10^7^ CFU/mL, for KPC-producer isolates, while the highest average concentration detected was 5.37 × 10^7^ CFU/mL, for OXA-48-producer isolates. The lowest bacterial concentration detected was 4.12 × 10^6^ CFU/mL, for a KPC–producer isolate. NDM, VIM, and OXA-48-producing isolates were detected at concentrations starting from 3.33 × 10^7^ CFU/mL.

## 4. Discussion

Infection with CRE increases the risk for prolonged hospitalization and mortality, and correlates directly with the development of septic shock and the use of incorrect empirical antibiotic therapy [6]. Moreover, mortality risk associated with CP-CRE infections is 3–6 times higher compared to that of infections caused by non-resistant bacteria [12]. The rising prevalence of CRE worldwide, along with the high potential for person-to-person transmission, present an urgent need for improved identification of CRE infections in order to reduce associated morbidity, mortality, and costs, and to minimize their spread.

In recent years, various assays have been developed for direct pathogen detection in patient blood, including bacterial antigen identification. The NG-Test CARBA 5 kit allows for detection of carbapenemases via a colorimetric assay, within 15 min. When first developed, it was shown to be a reliable diagnostic tool for recognition of carbapenemases in isolated bacterial colonies. A recent study that evaluated the kit’s performance in direct detection of carbapenemase-producing organisms in positive blood cultures, demonstrated an overall sensitivity and specificity of 98.3% and 100%, respectively [10]. When the kit was used exclusively to test clinical BCs and to test only for KPC, VIM, and OXA-48-like carbapenemases, it exhibited a sensitivity of 98.1% and specificity of 100%. Additionally, the current study tested isolates with all five types of carbapenemases, while the former study included isolates with either KPC, VIM, or OXA-48 carbapenemases.

The PPV and NPV determined in the present work were 100% and 66.7%, respectively, which aligned with that reported for the Resist-3 O.K.N. immunochromatographic lateral flow test (ICT) (Coris BioConcept, Gembloux, Belgium), an immunochromatographic assay designed to detect OXA-48-like, KPC, and NDM carbapenemases [13].

Another work that tested the performance of the NG-Test CARBA 5 assay with spiked BC samples, reported sensitivity and specificity of 97.7% and 96.1%, respectively [11]. While the present work failed to demonstrate a similar overall sensitivity, maximum specificity was 100%. It should be noted that, in the current study, the simulated BCs were not loaded into a blood culture incubator, while the above-mentioned studies used a blood culture incubator and tested the blood sample for CRE only after an alert of positivity was made by the incubator. This difference in the experimental setup may have resulted in low bacterial concentrations in the current study, that led to the lower sensitivity of CRE detection, as compared to other studies.

The lowest concentration of KPC-producing CRE recognized by the NG-Test CARBA 5 assay was 4.12 × 10^6^ CFU/mL, while for CREs expressing other carbapenemases, the lowest identified concentration was 3.33 × 10^7^ CFU/mL. In contrast, the BactAlert blood culture system (bioMérieux) required bacterial concentrations of 5 × 10^7^–5 × 10^9^ CFU/mL to determine positivity of blood cultures [14].

The limitations of this work included the use of spiked BCs; future work will be required to establish the assay’s performance in a real-world clinical laboratory setting. In addition, failure to identify the IMP producer may require an improved protocol, as recommended by the work of Volland and colleagues [15].

To conclude, this study demonstrated the reliability of the NG-Test CARBA 5 kit in rapid diagnosis of CRE bloodstream infections. When compared to molecular kits for detection of CRE, such as the Xpert Carba-R PCR assay, the time-to-result of the NG-Test CARBA 5 kit is less than 30 min, while that of the PCR assay is one hour. Additionally, the phenotypic kit is significantly cheaper. Given these advantages, we believe that the NG-Test CARBA 5 kit will soon be implemented in the laboratory for detection of CRE in blood culture.

## Figures and Tables

**Table 1:** Distribution of the different bacterial isolates by sample source

Sample Source	KPC (13)	NDM (10)	OXA-48 (8)	VIM (6)	IMP (1)	Non-CP (10)
Clinical						
Blood (6)	2	0	2	0	0	2
Other body fluids (6)	1	3	0	1	0	1
Urine (6)	1	1	1	0	0	3
Wounds (6)	2	0	1	1	1	1
Screening (Rectal samples)	7	6	4	4	0	3

**Table 2:** Distribution of the different bacterial isolates by carbapenem-resistance mechanism.

	Carbapenemase	NDM	OXA-48-Like	KPC	VIM	IMP	Non-CP
Bacterial Isolate							
*Citrobacter* spp. (*n* = 2)				2			
*Enterobacter aerogenes* (*n* = 2)					2		
*Enterobacter cloacae* (*n* = 8)		4		3	1		
*Enterobacter* spp. (*n* = 4)		2		1			1
*Enterococcus* spp. (*n* = 1)				1			
*Escherichia coli* (*n* = 8)			4		2	1	1
*Escherichia hermannii* (*n* = 2)				2			
*Klebsiella oxytoca* (*n* = 1)				1			
*Klebsiella pneumoniae* (*n* = 19)		3	4	3	1		8
*Providencia stuartii* (*n* = 1)		1					
Total (*n* = 48)		10	8	13	6	1	10

**Table 3:** Identification accuracy of carbapenem-resistant Enterobacterales (CRE) isolate resistance mechanism.

Carbapenemase Type (*N*)	Correct Identification *N* (%)	False Negative *N* (%)	Agreement Level (%)
KPC (13)	13 (100)	0 (0)	100
NDM (10)	8 (80)	2 (20)	80
OXA-48 (8)	7 (87.5)	1 (12.5)	87.5
VIM (6)	5 (83.3)	1 (16.6)	83.3
IMP (1)	0 (0)	1 (100)	0
Non-CP (10)	8 (100)	0 (0)	100
Total (48)	43 (89.5)	5 (10.5)	89.5

**Table 4:** Performance of the NG-CARBA 5 kit in detection of CRE in blood culture.

Measure	Value (%)
Agreement level	89.6
Sensitivity ^1^	86.84 (71.51–95.59)
Specificity ^1^	100 (69.15–100)
Positive predictive value (PPV) ^1^	100 (89.42–100)
Negative predictive value (NPV) ^1^	66.7 (38.38–88.18)

^1^ Values are presented in percentages, 95% confidence interval is shown in parentheses.

**Table 5:** Average and lowest bacterial concentrations detected by the NG-Test CARBA 5 kit, by carbapenemase type.

Type of Carbapenemase	Average Bacterial Concentration Detected (CFU/mL)	Lowest Bacterial Concentration Detected (CFU/mL)
NDM	5.12 × 10^7^	3.33 × 10^7^
KPC	3.57 × 10^7^	4.12 × 10^6^
OXA-48	3.79 × 10^7^	3.33 × 10^7^
VIM	5.37 × 10^7^	3.33 × 10^7^

## Data Availability

Data is contained within the article.
